# Environmental correlates of physical activity in multiple sclerosis: A cross-sectional study

**DOI:** 10.1186/1479-5868-4-49

**Published:** 2007-10-08

**Authors:** Shawna E Doerksen, Robert W Motl, Edward McAuley

**Affiliations:** 1 Department of Kinesiology and Community Health, University of Illinois at Urbana-Champaign, Urbana, USA

## Abstract

**Background:**

Multiple sclerosis (MS) is a chronic neurological disease that is associated with physical inactivity. Understanding the factors that correlate with physical activity is important for developing effective physical activity promotion programs for this population. Thus, we conducted a cross-sectional study that examined the association between features of the built environment with self-reported and objectively measured physical activity behaviour in adults with MS.

**Methods:**

Participants with MS (n = 196) were sent a questionnaire packet that included self-report measures of the built environment and physical activity and a pedometer in the mail and were instructed to complete the questionnaires and wear the device for seven days. Participants returned the completed questionnaires in a pre-stamped, pre-addressed envelope. Bivariate correlation analysis was conducted for examining associations between items on the environmental questionnaire with the two measures of physical activity. Stepwise regression analysis was conducted for determining the independent contributions of the significant environmental correlates for explaining variation in physical activity.

**Results:**

Correlational analysis indicated that presence of shops, stores, markets or other places within walking distance (r = .20; ρ = .18), presence of a transit stop within walking distance (r = .20; ρ = .16), and accessibility of free or low-cost recreation facilities (r = .16; ρ = .15) were related to pedometer, but not self-reported, measured physical activity. Regression analysis indicated that the presence of a transit stop within walking distance independently explained 4% of variance in pedometer measured physical activity.

**Conclusion:**

Physical activity is an important behaviour to promote among individuals with MS. This study indicated that aspects of the built environment are related to this health promoting behaviour among those with MS. Further research should focus on the longitudinal relationships among aspects of the environment with physical activity so as to provide strong background for developing effective promotion programs for people with MS.

## Background

Multiple sclerosis (MS) is a chronic and unpredictable neurological disease that affects an estimated 2 million people worldwide and 400,000 people in the United States [1]. The majority of people with MS are diagnosed between 20 and 50 years of age, and women are affected more often than men [1]. MS is more common among people with northern European ancestry than people of African, Asian, and Hispanic backgrounds [1].

MS involves demyelination and transection of the axons of neurons in the brain, brain stem, optic nerve, and spinal cord. The demyelination and transection interfere with the smooth and rapid conduction of electrical potentials along neuronal pathways in the central nervous system. This interference is associated with the diverse symptoms experienced by people with MS [1]. The disease process can result in mobility limitations, disability, and reduced quality of life. For example, 50% of individuals will require the use of an aide for walking and 10% will require a wheelchair within 15 years after the onset of MS [2,3].

To date, there is no effective mode of preventing MS. Therefore, researchers and clinicians have examined methods for slowing the progression of MS and managing its symptoms. Physical activity is an important behavioural factor for improving and managing the physical demands of MS [4,5] and has positive influences on symptom management through effects on fatigue, spasticity, mobility, depression, and pain [1,6]. Nevertheless, individuals with MS are relatively inactive compared with non-diseased populations [7,8]. The rate of inactivity in MS is alarming given the accepted prevalence of sedentary lifestyles among healthy and non-diseased adults in the United States [9].

Very little research has examined correlates of physical activity among individuals with MS. Such research is important for understanding modifiable and non-modifiable influences on physical activity behaviour in MS. We are aware of four published studies that have examined correlates of physical activity among those with MS [10-13]. The studies generally focused on personal and psychological factors as correlates of physical activity in individuals with MS. For example, one study reported that individuals who perceived themselves as more able to perform physical activity and having less severe impairment reported higher levels of physical activity [13]. Another study examined perceived barriers as a correlate of physical activity in MS patients who lived in urban and rural regions and found that those living in rural areas were less active than their urban-dwelling counterparts [12]. Further, perceived barriers (e.g., lack of time) were correlated with physical activity in MS patients living in either urban or rural environments, even after controlling for disability.

The physical environment, either actual or perceived, might represent an important correlate of physical activity in persons with MS, but it has been relatively unexamined in this population. Aspects of the physical environment have been linked with physical activity behaviour in healthy, non-diseased populations. One recent meta-analysis reported that accessibility of physical activity facilities, sidewalks, presence of shops and services, and lack of traffic were significant correlated with physical activity participation in non-diseased adults [14]. Important caveats noted in that meta-analysis involved the reliance upon self-report rather than objective measures of physical activity and lack of examinations of environmental correlates beyond non-diseased populations. One might expect that accessibility of facilities and transportation, presence of shops and services, and lack of traffic would be particularly important correlates of physical activity in individuals with disease such as MS that profoundly affects ambulation and mobility.

This study involved an examination of perceived environmental variables as correlates of self-reported and objectively measured physical activity in individuals with MS. We hypothesized that environmental characteristics such as presence of shops and services, transportation, and accessible facilities would exhibit small-to-moderate correlations with physical activity in individuals with MS, and that the associations would be stronger for the objective than self-report measure of physical activity.

## Methods

### Participants

We recruited a convenience sample of individuals with MS using media advertisements that were distributed by the Greater Illinois, Indiana, and Gateway chapters of the National Multiple Sclerosis Society; those chapters collectively serve an estimated 30,000 people with MS. Individuals who were interested in participating initially contacted the research team through either e-mail or telephone. This was followed-up by a telephone call from a member of the research team who described the study and its procedures, answered all questions, and conducted a brief screening of inclusion criteria: (a) having an established definite diagnosis of MS, (b) being relapse free during the last 30 days, and (c) being ambulatory with minimal assistance (i.e., walking with or without a cane). There were 221 individuals who underwent the screening, and 25 individuals, or 11%, did not satisfy our inclusion criteria and were excluded from participation. The final sample included 196 individuals with MS. There were 174 individuals diagnosed with relapsing-remitting MS and 22 individuals diagnosed with primary or secondary progressive MS. The mean time since diagnosis of MS was 9.0 years (SD = 7.1) and the mean age of the sample was 46.1 years (SD = 9.8). The sample was primarily female (88%), Caucasian (93%), and well educated (60% had 1 or more years of college education) with a median annual household income exceeding $40,000 (65%).

### Procedure

The procedure for this study was approved by an Institutional Review Board, and all participants provided written informed consent. After initial telephone contact and voluntary agreement of participation, the informed consent document, battery of paper-and-pencil questionnaires, and pedometer were sent to each participant through the United States Postal Service (USPS). The researchers called to make sure the participants received the package, understood the directions, and signed the informed consent document. The participants were instructed to complete the battery of questionnaires and then to wear the pedometer during the waking hours, except while showering, bathing, and swimming, for a seven-day period. Waking hours were defined as the moment upon getting out of bed in the morning through the moment of getting into bed in the evening. The participant recorded the time that the pedometer was worn as well as daily step counts on a log. After completing the measures and wearing the pedometer for seven days, participants returned a signed copy of the informed consent document and the study materials in a pre-stamped, pre-addressed envelope through the USPS. Each battery of questionnaires was checked for completeness and participants were called to collect missing data. Each participant received $20 remuneration and a complementary pedometer.

### Measures

#### Physical activity

Physical activity was measured using the short-form of the International Physical Activity Questionnaire (IPAQ) [15] and a Yamax SW-200 pedometer (Yamax Corporation, Tokyo, Japan). The short-form of the IPAQ was designed as an instrument for population surveillance of physical activity among adults. The short-form of the IPAQ contains six items that measure the frequency and duration of vigorous-intensity activities, moderate-intensity activities, and walking during a seven-day period. The respective frequency and duration values for vigorous, moderate, and walking activities are initially multiplied, and the resulting volumes of vigorous, moderate, and walking activities are multiplied by 8, 4, and 3.3 metabolic equivalents, respectively, and then summed to form a continuous measure of physical activity in MET-minutes/week. The short-form of the IPAQ includes an additional question that measures time spent sitting as an indicator of sedentary activity and that is not included as part of the summary physical activity score. This study did not include the sitting item in the data analyses because we were not interested in sedentary behaviour. Hence, the primary variables included in the data analysis were total IPAQ METs, Walking METs, and Moderate-to-vigorous physical activity METs. The Yamax SW-200 pedometer (Yamax Corporation, Tokyo, Japan) was used in our study because this brand has received the most scientific attention in non-diseased populations. The Yamax SW-200 pedometer digitally displays step counts. Each pedometer was checked for accuracy using a brief walking test (500 steps at 80 m·min^-1 ^on a treadmill), and if the error exceeded 1% (i.e., 5 steps) the pedometer was not used in this study. All pedometers passed the walking test and an additional shake test. Participants recorded the pedometer step counts in a log on a daily basis, and the daily counts were averaged across a 7-day period and this average was used in data analysis.

#### Perceived environment

The perceived environment was measured using the Environment module of the IPAQ (IPAQ-E). The IPAQ-E includes seven core items, four recommended, and six optional items. We only included the seven core and four recommended items. The seven core items measure the structural components of the environment including the type of housing in a person's neighbourhood along with items that pertain more to walking and bicycling behaviours such as the presence of sidewalks, amenities, and recreation facilities. The four recommended items measure other related components of the environment including traffic and aesthetics. The items for the IPAQ-E were adapted from two previously published measures [16,17] and the IPAQ-E has recently been used in studies of both adults and adolescents [18-20]. There is recent preliminary evidence supporting the reliability and validity of IPAQ-E scores in a random sample of adults [21].

### Data analysis

Analyses were based on a sample of 195 individuals as one participant provided only one day of pedometer data. Of the remaining participants, two individuals provided six days worth of data whereas the rest of the sample (n = 193) provided a total of seven days. Descriptive data are presented as mean score ± standard deviation. The relationships between scores from the measures of physical activity with scores from the IPAQ-E were analyzed using Pearson product-moment correlations in SPSS, version 14 (SPSS, Chicago, IL). We further examined the relationships using Spearman correlations (ρ), which provide a non-parametric version of a Pearson correlation coefficient on the basis of ranks of the data rather than the actual values. This is important as physical activity data can be positively skewed, and the Spearman correlations do not rely on the assumption of normality. Cohen's [22] guidelines of .1, .3, and .5 were used for judging the magnitude of the correlations as small, moderate, and large, respectively. Stepwise multiple regression analysis with a forward entry was then conducted to determine the independent contributions of the significant variables on the IPAQ-E for explaining variation in physical activity.

## Results

The average daily step count for the overall sample was 5,887 steps per day (SD = 3,226) with a range of 1,190 to 19,473 steps per day. The large range is important because it permits an examination of environmental variables as correlates of physical activity. The average daily step counts for the overall sample can be interpreted based on preliminary pedometer-determined physical activity cut points for healthy adults of <5,000 steps/day as sedentary, 5,000–7,499 steps/day as low active, 7,500–9,999 steps/day as somewhat active, 10,000–12,499 steps/day as active, and >12,500 steps/day as highly active [23]. This would suggest that the present sample was generally engaging in a low amount of physical activity.

The correlation coefficients are provided in Table 1. There were three items on the IPAQ-E (items 2, 3, and 6) that were significantly correlated with average daily step counts from the pedometers; no items on the IPAQ-E were significantly correlated with total MET-minutes/week, walking MET-minutes/week, or MET-minutes/week in moderate-to-vigorous activity scores from the short form of the IPAQ. Item 2 involved the presence of shops, stores, markets, or other places within easy walking distance and Item 3 involved the presence of a transit stop within a 10–15 minute walk from one's home. Item 6 involved accessibility of free or low-cost recreation facilities including the presence of parks, walking trails, bike paths, and playgrounds.

**Table 1:** Correlations among self-report and pedometer measures of physical activity and environmental variables.

	*1*	*2*	*3*	*4*	*5*	*6*	*7*	*8*	*9*	*10*	*11*	*12*	*13*	*14*	*15*
*1. IPAQ_MET*	__														
*2. IPAQ_WALK*	.77‡ (.76‡)	__													
*3. IPAQ_MVPA*	.91‡ (.83‡)	.44‡ (.37‡)	__												
*4. Pedometer*	.32‡ (.30‡)	.23‡ (.30‡)	.30‡ (.27‡)	__											
*5. Dwelling density*	-.11 (-.12)	-.07 (-.07)	-.11 (-.12)	-.05 (-.05)	__										
*6. Access to ammenities*	.10 (.06)	.02 (.04)	.13 (.10)	.20‡ (.18†)	.07 (.05)	__									
*7. Access to transit stops*	.01 (.01)	-.03 (-.02)	.03 (.01)	.20‡ (.13†)	.13 (.18)	.46‡ (.48‡)	__								
*8. Presence of sidewalks*	.05 (.05)	.08 (.08)	.02 (-.01)	.09 (.04)	.15† (.14†)	.23‡ (.19‡)	.35‡ (.35‡)	__							
*9. Presence of bicycle facilities*	.05 (.04)	.08 (.01)	.01 (.04)	.06 (.02)	.07 (.06)	.24‡ (.23‡)	.21‡ (.22‡)	.38‡ (.38‡)	__						
*10. Presence of low cost recreation*	.03 (.04)	.04 (.07)	.02 (.01)	.16† (.15†)	.03 (.02)	.28‡ (.27‡)	.28‡ (.28‡)	.31‡ (.30‡)	.63‡ (.62‡)	__					
*11. Level of crime*	-.05 (-.09)	-.04 (-.03)	-.05 (-.11)	-.04 (-.08)	.26‡ (.25‡)	.19‡ (.21‡)	.21‡ (.21‡)	.11 (.11)	-.01 (-.01)	-.02 (.00)	__				
*12. Presence of heavy traffic*	.12 (.11)	.06 (.04)	.13 (.13)	.05 (.03)	.10 (.11)	.14† (.18†)	.21‡ (.21‡)	-.10 (-.09)	-.03 (-.04)	.00 (-.02)	.36‡ (.37‡)	__			
*13. Presence of active others*	.01 (.02)	.03 (-.02)	-.01 (.02)	.12 (.10)	.10 (.07)	.20‡ (.18†)	.20‡ (.19‡)	.20‡ (.18†)	.34‡ (.35‡)	.34‡ (.34‡)	-.06 (-.05)	-.13† (-.12)	__		
*14. Aesthetics of neighbourhood*	-.06 (-.01)	-.04 (.03)	-.06 (-.03)	-.02 (-.01)	.02 (-.01)	.02 (.03)	.03 (.04)	.07 (.08)	.22‡ (.23‡)	.15† (.16†)	-.19‡ (-.17†)	-.14† (-.16†)	.35‡ (.34‡)	__	
*15. Number of motor vehicles at home*	.10 (.07)	.08 (.01)	.09 (.09)	-.07 (.01)	-.25‡ (-.29‡)	-.32‡ (-.33‡)	-.29‡ (-.26‡)	-.23‡ (-.20‡)	-.15† (-.14)	-.14† (-.11)	-.17‡ (-.16†)	-.06 (-.10)	-.04 (-.02)	.01 (-.01)	__

†p < .05; ‡p < .005

Note: Values are reported as Pearson correlations, r, first, followed by Spearman correlations, (ρ). Further, numbers 5–14 within the matrix refer to items 1–11 of the IPAQ-Environment module.

The next analysis involved regressing average daily step counts from the pedometers on Items 2, 3, and 6 from the IPAQ-E. The model was statistically significant, *F*(1,194) = 8.13, *p *= .005, and accounted for 4% of the variance in daily step counts from the pedometer (adjusted *R*^2 ^= .04). The only variable that entered into the regression model that was found to be significant was Item 3 (β = .20) such that presence of a transit stop within a 10–15 minute walk of one's home was positively associated with pedometer counts. The adjusted *R*^2 ^value of .04 translates into an *f*^2 ^value of .04 based on the formula, *f*^2 ^= *R*^2^/(1 - *R*^2^). The *f*^2 ^value of .04 is interpreted as a small effect size based on guidelines of .02, .15, and .35 as small, moderate, and large, respectively [22].

## Discussion

The present study involved an examination of environmental variables as correlates of self-reported and objectively measured physical activity in individuals with the chronic and unpredictable neurological disease of MS. Our results indicated that presence of shops and stores, accessibility of public transportation within a 10–15 minute walk from one's home, and presence of free or low-cost recreation facilities were all associated with the objective, but not self-report, measure of physical activity. Additional analyses indicated that the accessibility of public transportation within a 10–15 minute walk from one's home was independently associated with objectively measured physical activity. The variables generally exhibited small-to-moderate correlations with physical activity and explained only a modest amount of variation (4%) in average daily step counts from the pedometer.

Our findings are remarkably consistent with the results of a recent meta-analysis of the association between environmental characteristics and physical activity. That meta-analysis identified accessibility of physical activity facilities, sidewalks, presence of shops and services, and lack of traffic as important correlates of physical activity participation in non-diseased adults [14]. We similarly reported that the presence of shops and stores and presence of free or low-cost recreation facilities were all associated with objectively measured physical activity in individuals with MS. Importantly, the present study did avoid a caveat noted in the meta-analysis by including both self-report and objective measures of physical activity and a limitation noted in the meta-analysis by extending the examination of environmental correlates beyond non-diseased populations into those with MS.

There is evidence that physical activity differs based on the area of residence in individuals with MS. Those individuals who lived in rural environments were reported to be less active than urban-dwellers [12]. However, there was a lack of examination of the elements within the urban environment that were associated with physical activity. The present study indicates that the presence of shops, services, and public transportation within walking distance and low cost/free facilities were significantly associated with physical activity behaviour. These types of environmental features are likely to be more prominent in an urban area than in a rural setting, and would bolster the argument that urban areas are better suited for physical activity participation and clarify the previous findings that physical activity differs based on the area of residence in individuals with MS. We believe that further study of the characteristics of urban and rural areas should be undertaken for identifying the variables that explain the residential-based difference in physical activity among persons with MS.

One important observation of this study was the differential pattern of relationship between environmental variables and the self-report and objective measures of physical activity. That is, there were significant associations between environmental variables and the objective measure of physical activity based on a pedometer, whereas there were no significant associations with the self-report measure based on the short form of the IPAQ. This is consistent with the notion that objective measures might provide a more precise measurement of physical activity than self-report measures and the recommendation that the study of environmental influences on physical activity should be undertaken using objective measurement technologies [14].

The strengths of this study include examining environmental characteristics as correlates of self-report and objectively measured physical activity in a sample of individuals with MS. This is important as one might expect that accessibility of physical activity facilities, presence of shops and services, and accessibility of public transportation would be particularly important correlates of physical activity in individuals with a chronic and unpredictable neurological disease such as MS that is associated with mobility and ambulatory impairments. Nevertheless, there are limitations to be taken into consideration with the present study. First, the data are cross-sectional in nature, and this does not allow for conclusions regarding the causal nature of the environmental influence on physical activity behaviour. However, the data do provide a sound initial basis for developing a research agenda regarding physical activity behaviour and the environment in those with MS. Second, our convenience sample primarily consisted of females who were Caucasian and well-educated and this may limit the generalisability of these results to the entire American MS population. Third, we did not include an objective measurement of environment, which would have added an interesting level of analysis. The measurement of the environment that we used focuses on the participant's perceptions of the environment. Obtaining measurements of these perceptions as well as an objective measure of the physical environment would allow us to determine if there are discrepancies between the two views of the environment. Further, this information may provide insight on how to modify perceptions so that they accurately reflect what exists within the actual environment.

Understanding the correlates of physical activity for those with MS will allow for the design and implementation of appropriate and effective physical activity interventions for those with MS. As physical activity is an effective method of symptom management [6,24], development of effective promotion programs for this population is imperative and should be of high value to the health care profession. The present findings outline several characteristics of the built environment that are related to physical activity and should be considered by health professionals and urban planners when designing programs or neighbourhoods in order to encourage physical activity behaviour among individuals with MS. Although these results provide insight into the correlates of physical activity in this population, further investigation of the predictors of this behaviour is warranted. Especially necessary is the examination of these constructs using a prospective design to determine which elements are essential for behaviour change. Once these are identified, further development of effective interventions can proceed.

## Conclusion

MS is a highly prevalent disease worldwide and in the American population. As there is currently no cure for MS, there is a need for identifying and promoting alternative treatment options. Physical activity has been shown to be an effective method of symptom management in MS, but there is a high rate of inactivity in this population. Therefore, determining the factors that are associated with this behaviour is important in order to appropriately promote physical activity. The built environment has been shown to be an important correlate of physical activity in healthy, non-diseased populations. Our data indicate that environmental qualities such as having amenities within walking distance, access to low cost facilities, and proximity to transit stops are associated with higher levels of activity in adults with MS. These findings are important as they are the first that point to specific characteristics of the environment that are associated with physical activity behaviour among those with MS. Further investigation of these environmental characteristics could provide a basis for effective interventions to promote physical activity in this population.

## Competing interests

The author(s) declare that they have no competing interests.

## Authors' contributions

SD drafted the manuscript.

RM conceived of the study, performed data analyses and assisted in drafting the manuscript.

EM assisted in drafting the manuscript.

All authors have read and approved the final draft of the manuscript.

## References

[B1] National Multiple Sclerosis Society (2003). Multiple Sclerosis Information Sourcebook.

[B2] Weinshenker BG, Bass B, Rice GP, Noseworthy J, Carriere W, Baskerville J, Ebers GC (1989). The natural history of multiple sclerosis: a geographically based study. I. Clinical course and disability. Brain.

[B3] Runmarker B, Andersen O (1993). Prognostic factors in multiple sclerosis incident cohort with twenty-five years of follow-up. Brain.

[B4] Simmons RD, Ponsonby A-L, van der Mei IAF, Sheridan P (2004). What affects your MS? Responses to an anonymous, internet-based epidemiological survey. Multiple Sclerosis.

[B5] Stuifbergen AK (1992). Meeting the demands of illness: Types and sources of support for individuals with MS and their partners. Rehabilitation and Nursing Research.

[B6] Schapiro RT (2003). Managing the Symptoms of Multiple Sclerosis.

[B7] Motl RW, Snook EM, McAuley E, Columbus F (2005). Physical activity and its correlates among people with multiple sclerosis: Literature review and future directions. Progress in Multiple Sclerosis Research.

[B8] Motl RW, Snook EM, McAuley E (2005). Physical activity and multiple sclerosis: a meta-analysis. Multiple Sclerosis.

[B9] Centers for Disease Control and Prevention (CDC) (2004). Prevalence of no leisure-time physical activity--35 States and the District of Columbia, 1988-2002.. MMWR Morb Mortal Wkly Rep.

[B10] Motl RW, Snook EM, McAuley E, Gliottoni RC (2006). Symptoms, self-efficacy, and physical activity among individuals with multiple sclerosis. Research in Nursing & Health.

[B11] Motl RW, Snook EM, McAuley E, Scott JA, Douglass ML (2006). Correlates of physical activity among individuals with multiple sclerosis. Annals of Behavioral Medicine.

[B12] Stuifbergen AK (1999). Barriers and health behaviors of rural and urban persons with MS. American Journal of Health Behavior.

[B13] Stuifbergen AK, Roberts GJ (1997). Health promotion practices of women with multiple sclerosis. Arch Phys Med Rehabil.

[B14] Duncan MJ, Spence JC, Mummery WK (2005). Perceived environment and physical activity: a meta-analysis of selected environmental characteristics. Int J Behav Nutr Phys Act.

[B15] Craig CL, Marshall AL, Sjöström M, Bauman AE, Booth ML, Ainsworth BE, Pratt M, Ekelund U, Yngve A, Sallis JF, Oja P (2003). International physical activity questionnaire: 12-country reliability and validity.. Med Sci Sports Exerc.

[B16] Addy CL, Wilson DK, Kirtland KA, Ainsworth BE, Sharpe P, Kimsey D (2004). Association of perceived social and physical environmental supports with physical activity and walking behavior. American Journal of Public Health.

[B17] Saelens BE, Sallis JF, Black JB, Chen D (2003). Neighborhood-based differences in physical activity: An environment scale evaluation. American Journal of Public Health.

[B18] Bengoechea EG, Spence JC, McGannon KR (2005). Gender differences in perceived environmental correlates of physical activity. Int J Behav Nutr Phys Act.

[B19] Mota J, Almeida M, Santos P, Ribeiro JC (2005). Perceived neighborhood environments and physical activity in adolescents. Preventive Medicine.

[B20] Weir LA, Etelson D, Brand DA (2006). Parents' perceptions of neighborhood safety and children's physical activity. Preventive Medicine.

[B21] Alexander A, Bergman P, Hagstromer M, Sjostrom M (2006). IPAQ environmental module: Reliability testing. Journal of Public Health.

[B22] Cohen J (1988). Statistical Power Analysis for the Behavioral Sciences.

[B23] Tudor-Locke C, Bassett DR (2004). How many steps/day are enough? Preliminary pedometer indices for public health. Sports Medicine.

[B24] Hamler B (2006). Exercises for Multiple Sclerosis: A Safe and Effective Program to Fight Fatigue, Build Strength, and Improve Balance.

